# A statistical approach for detecting genomic aberrations in heterogeneous tumor samples from single nucleotide polymorphism genotyping data

**DOI:** 10.1186/gb-2010-11-9-r92

**Published:** 2010-09-21

**Authors:** Christopher Yau, Dmitri Mouradov, Robert N Jorissen, Stefano Colella, Ghazala Mirza, Graham Steers, Adrian Harris, Jiannis Ragoussis, Oliver Sieber, Christopher C Holmes

**Affiliations:** 1 Department of Statistics, University of Oxford, South Parks Road, Oxford, OX1 3TG, UK; 2 Ludwig Colon Cancer Initiative Laboratory, Ludwig Institute for Cancer Research, Royal Melbourne Hospital, Victoria 3050, Australia; 3 Wellcome Trust Centre for Human Genetics, University of Oxford, Roosevelt Drive, Oxford, OX3 7BN, UK; 4 Molecular Oncology Laboratories, Department of Medical Oncology, University of Oxford, Weatherall institute of Molecular Medicine, Headington, Oxford OX3 9DS, UK; 5 MRC Harwell, Harwell Science and Innovation Campus, Oxfordshire, OX11 0RD, UK; 6 Current Address: UMR203 INRA INSA-Lyon BF2I, Biologie Fonctionnelle Insectes et Interactions, Bat. L. Pasteur, 20 ave. A. Einstein, F-69621 Villeurbanne Cedex, France

## Abstract

We describe a statistical method for the characterization of genomic aberrations in single nucleotide polymorphism microarray data acquired from cancer genomes. Our approach allows us to model the joint effect of polyploidy, normal DNA contamination and intra-tumour heterogeneity within a single unified Bayesian framework. We demonstrate the efficacy of our method on numerous datasets including laboratory generated mixtures of normal-cancer cell lines and real primary tumours.

## Background

Single nucleotide polymorphism (SNP) genotyping microarrays provide a relatively low-cost, high-throughput platform for genome-wide pro ling of DNA copy number alterations (CNAs) and loss-of-heterozygosity (LOH) in cancer genomes. These arrays have enabled the discovery of genomic aberrations associated with cancer development or prognosis [1-4] and two recent studies, in particular, have examined 746 cancer cell lines [5] and 26 cancer types [6] revealing much about the landscape of the cancer genome. However, whilst numerous robust computational methods are available for the detection of copy number variants (CNVs) in normal genomes [7-11]; the approaches applied to cancers are often sub-optimal due to data properties that are unique or more pronounced in cancer.

Potential difficulties in the analysis of SNP data from cancers have been considered since the earliest SNP array based cancer studies [12-14] with the principle obstacles being (1) variable tumor purity (normal DNA contamination), (2) intra-tumor genetic heterogeneity, (3) complex patterns of CNA and LOH events, and (4) genomic instability leading to aneuploidy/polyploidy. Moreover, these issues are also confounded by previously well-described technical artifacts associated with SNP arrays such as: signal variation due to local sequence content [15] and, complex noise patterns due to variable sample quality and experimental conditions [16].

Dedicated cancer analysis tools that compensate for some of these factors have recently begun to emerge [17-27] but there is currently no single coherent statistical model-based framework that unifies and extends all the principles underlying these many methods. Here, we propose such a framework and illustrate, on a number of different datasets, the improvements in terms of robustness and versatility that can be gained in cancer genome pro ling, particularly in large-sample cancer studies involving the investigation of different molecular sub-types and the use of modern high-resolution SNP arrays (greater than 500,000 markers). Our methods are implemented in a piece of software we call OncoSNP.

### Characteristics of SNP data acquired from cancer genomes

We begin with a brief examination of the characteristics of SNP array data acquired from cancer genomes (for a more thorough review of SNP array analysis and methodology, see [28-31]). SNP array analysis produces two types of summary measurement for each SNP probe: (i) the Log R Ratio (LRR) which is a measure related to total copy number, analogous to the log ratio in array comparative genomic hybridization (aCGH) experiments; and (ii) the B allele frequency (BAF), which measures the relative contribution of the B allele to the total signal (here we use A and B as generic labels to refer to the two alternative SNP alleles). Normalization methods to extract these measurements for the Illumina and Affymetrix SNP genotyping platforms have been previously described [32,33] but is not a subject we treat in detail in this article. In this paper, our examples are based on the Illumina platform and we primarily use the default normalization offered by Illumina's proprietary BeadStudio/GenomeStudio software or the tQN normalization [33] where appropriate. However, the methods described are not intrinsically tied to the Illumina platform and we are actively working to transfer these techniques for use with the Affymetrix platform.

**Figure 1:**
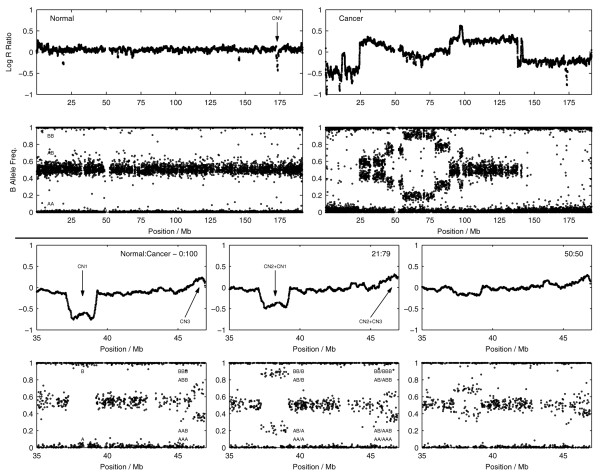
**Example cancer SNP data**. (Top panel) SNP data showing the distribution of Log R Ratio (LRR) and B allele frequencies (BAF) values across chromosome 1 for a cancer cell line (HCC1395) and its matched normal (HCC1395BL). The normal sample is characterized by a typical diploid pattern of zero mean LRR (copy number 2) and BAF values distributed around 0, 0.5 and 1 (genotypes AA, AB and BB) with occasional aberrations due to copy germline number variants (CNV). The cancer cell line consists of complex patterns of LRR and BAF values due to a variety of copy number alterations and loss-of-heterozygosity events. (Bottom panel) SNP data is shown for a single copy deletion and duplication on chromosome 21 for various normal-cancer cell line dilutions. In the presence of normal DNA contamination, the LRR signals for the deletion and duplication are diminished in magnitude and the distribution of the BAF values reflects the aggregated effect of mixed normal and cancer genotypes at each SNP. Note - the Log R Ratio values are smoothed and thinned for illustrative purposes.

Figure 1 (top panel) depicts data for chromosome 1 of a breast cancer cell line (HCC1395, ATCC CRL-2324) and a EBV transformed lymphoblastoid cell line (HCC1395BL, ATCC CRL-2325) derived from the same patient from a previously published dataset [24]. Downward shifts in the Log R Ratios indicate DNA copy number losses relative to overall genome dosage, whilst copy number gains cause upward shifts. The BAF tracks changes in the relative fractions of the B allele due to CNA and/or LOH.

In the non-cancer (normal) lymphoblastoid cell line, the LRRs are distributed around zero corresponding to DNA copy number 2; whilst the BAFs are clustered around values of 0, 0.5 and 1 that correspond to the diploid genotypes AA, AB and BB. Small aberrations in the normal data can be observed due to germ line CNVs but the genome is otherwise stable. The cancer cell line presents a much more complex scenario with extensive genomic rearrangements leading to considerable variation in the SNP data. This is not an atypical scenario for cancers which often feature large numbers of focal aberrations and whole or partial chromosomal copy number changes although this can vary considerably depending on the cancer type and the stage of the disease. The question we address here is: how do we translate this SNP data into actual copy number and LOH calls?

### Effects of polyploidy

One distinctive difference between the normal and cancer datasets is that the LRR values are not directly comparable. Experimental protocols for SNP arrays constrain the amount of DNA, not the number of cells, to be the same for each sample assayed. For example, a purely metalloid genome containing no other chromosomal alterations could not be distinguished from a diploid genome, as the same mass of genomic material would be hybridized on to the SNP array. The situation is further compounded by standard normalization methods that transform the probe intensity data on to a common reference scale or "virtual diploid state" [34] in order to correct for between-array or cross-sample variability.

The result is that the (zero) baseline of the LRR for the cancer cell line or tumor sample does not correspond to a normal diploid copy number but to the average copy number (ploidy) of the sample. In order to determine absolute copy number values, a correct baseline for the interpretation of the LRR values must be determined but this is a challenging problem since, for any particular cancer sample, the ploidy is generally unknown a priori, maybe a fractional value and varies from one cancer to the next. Methods to tackle baseline uncertainty for polyploid tumors have recently been developed [17,21] but these are only effective in the absence of normal DNA contamination and intra-tumor heterogeneity making them most effective for use with cancer cell lines and very high purity tumor samples.

### Normal contamination and intra-tumor heterogeneity

Normal DNA contamination can also be a significant barrier to the correct interpretation of SNP data as illustrated in Figure 1 (bottom panel). The SNP data shown comes from various artificial mixtures of the cancer cell line and paired normal cell line [33] for a single-copy deletion and duplication on chromosome 21. The SNP array measures both the contribution of the normal and tumor genotypes hence, the B allele frequencies for the deletion and duplication appear as four bands, ref1ecting the mixed normal-tumour genotypes AA/A, AB/A, AB/B or BB/B for the single-copy deletion and AA/AAA, AB/AAA, AB/BBB or BB/BBB for the single-copy duplication. Moreover, as the normal DNA content increases, the magnitude of the shifts in the LRR values associated with the deletion and duplication are reduced.

It is of interest to note that whilst the presence of normal DNA affects SNP data globally, localized variation can also exist due to intra-tumor heterogeneity and aggregation from multiple co-existing cancer cell clones each harboring their own distinct pattern of genomic aberrations. These mixed signals must be deconvolved in order to ascertain the underlying somatic changes and a number of methods [20,22,24-27] have been proposed to tackle the issue of normal DNA contamination. These approaches often assumed the absence of the effects of polyploidy described previously and therefore are principally suited to the analysis of normal DNA contaminated and near-diploid tumor samples.

## Results and Discussion

### Model overview

**Figure 2:**
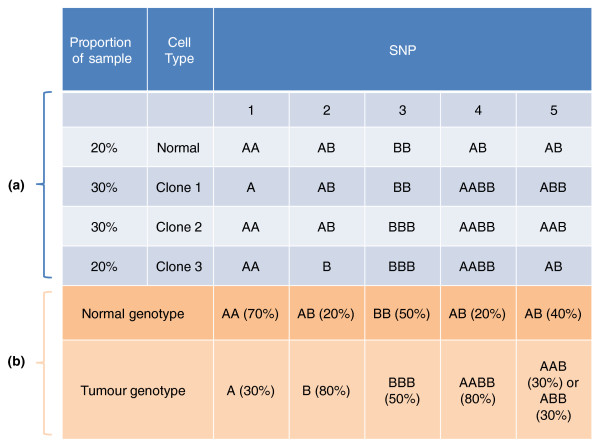
**Illustrating the statistical model**. **(a) **The tumor sample consists of DNA contributions from an unknown number of clones (here, we illustrate three clones) and normal cells in different proportions. Each clone has its own set of tumor genotypes which are derived from the normal genotypes by the loss or duplication of alleles. **(b) **Our statistical model assumes that, at each locus, there exists a normal and a common tumor genotype. OncoSNP estimates the normal and common tumor genotype and the proportion of the sample explained by each genotype from the SNP data. The situation depicted at SNP 5 involves clones with different tumor genotypes - this is not considered under our model.

The development of our method, implemented in OncoSNP, has been motivated by the need to address both the effects of normal DNA contamination and polyploidy simultaneously. Normal tissue contaminated polyploid tumors are frequently observed in studies of, for example, colon or breast cancers and, at the time of writing, only one method Genome Alteration Print [23], based on pattern recognition heuristics, has been developed to manage both these highly important issues in SNP array based cancer analysis. Our approach differs from previous methods in that it attempts to tackle the issues of normal DNA contamination, intra-tumor heterogeneity and baseline ploidy normalization artifacts jointly within a coherent statistical framework. The model assumes that, at each SNP, each tumor cell of a given specimen either retains the normal constitutional genotype or possesses an alternative but, common, tumor genotype. However, in contrast to other methods, we explicitly parameterize the proportion of cells that possess the normal genotype at each SNP. This proportion is determined by a genome-wide fraction attributed to normal DNA contamination and the proportion of tumor cells that have remained unchanged at that SNP which is allowed to vary along the genome thus allowing for intra-tumor heterogeneity (the underlying statistical model is illustrated in Figure 2). We also include a LRR baseline adjustment parameter that allows inference of the unknown tumor ploidy in a statistically rigorous manner.

Bayesian methodology is applied to impute the unknown normal-tumor genotypes, the normal genotype proportion and to assign a probabilistic score of each SNP belonging to one of twenty-one different "tumor states" (Table 1). Experimental noise is accounted for using a flexible semi-parametric noise (mixture of Student *t*-distributions) model that is able to adaptively fit complex noise distributions to the SNP data, and our method further adjusts for wave-like artifacts correlated to local GC content [35].

**Table 1:** OncoSNP tumor states

Tumor states			
**Tumor state**	**Tumor copy number**	**Allowable tumor-normal genotypes**	**Description**

1	0	(-, AA), (-, AB), (-, BB)	Homozygous deletion
2	1	(A, AA), (A, AB), (B, AB), (B, BB)	Hemizygous deletion
3	2	(AAAA, AA), (AAAB, AB), (ABBB, AB), (BBBB, BB)	Normal
4	3	(AAA, AA), (AAB, AB), (ABB, AB), (BBB, BB)	Single copy duplication
5	4	(AAAA, AA), (AAAB, AB), (ABBB, AB), (BBBB, BB)	4n monoallelic amplification
6	4	(AAAA, AA), (AABB, AB), (BBBB, BB)	4n balanced amplification
7	5	(AAAAA, AA), (AAAAB, AB), (ABBBB, AB), (BBBBB, BB)	5n monoallelic amplification
8	5	(AAAAA, AA), (AAABB, AB), (AABBB, AB), (BBBBB, BB)	5n unbalanced amplification
9	6	(AAAAAA, AA), (AAAAAB, AB), (ABBBBB, AB), (BBBBBB, BB)	6n unbalanced amplification
10	6	(AAAAAA, AA), (AAAABB, AB), (AABBBB, AB), (BBBBB, BB)	6n unbalanced amplification
11	6	(AAAAAA, AA), (AAABBB, AB), (BBBBB, BB)	6n unbalanced amplification
12	2	(AA, AA), (AA, AB), (BB, AB), (BB, BB)	2n somatic LOH
13	3	(AAA, AA), (AAA, AB), (BBB, AB), (BBB, BB)	3n somatic LOH
14	4	(AAAA, AA), (AAAA, AB), (BBBB, AB), (BBBB, BB)	4n somatic LOH
15	5	(AAAAA, AA), (AAAAA, AB), (BBBBB, AB), (BBBBB, BB)	5n somatic LOH
16	6	(AAAAAA, AA), (AAAAAA, AB), (BBBBBB, AB), (BBBBBB, BB)	6n somatic LOH
17	2	(AA, AA), (BB, BB)	2n germline LOH
18	2	(AAA, AA), (BBB, BB)	3n germline LOH
19	2	(AAAA, AA), (BBBB, BB)	4n germline LOH
20	2	(AAAAA, AA), (BBBBB, BB)	5n germline LOH
21	2	(AAAAAA, AA), (BBBBBB, BB)	6n germline LOH

Description of the 21 tumor states showing corresponding copy numbers and genotypes. OncoSNP assigns a score of each SNP being in each of the twenty-one tumor states.

Our MATLAB implementation typically requires between 0.5-3 hours processing per sample dataset (containing approximately 600,000 probes) depending on the run-time options specified. A variety of user settings are provided to allow the performance of the method to be tuned to the particular application and longer processing times are required where little prior information is provided and the method is required to learn all characteristics directly from data. As the method analyzes each sample independently, parallel processing of multiple samples simultaneously is trivially implemented.

### Polyploidy correction

In order to demonstrate the ability of OncoSNP to correctly adjust the baseline for the Log R Ratio to the actual baseline for aneuploid/polyploid samples, we analyzed SNP data for ten well-characterized cancer cell lines (Table 2). Karyotype information for each cell line were retrieved from the online database for the American Type Culture Collection (ATCC) or previous karyotype studies [36,37].

**Table 2:** Cancer cell lines

Cancer cell lines		
**Cell line**	**Chromosome number (modal, range)**	**Reference**

HL60	46 (44-46)	Liang et al. (1999)
HT29	70 (69-73)	Adbel-Rahman et al. (2000)
SW1417	70 (66-71)	Adbel-Rahman et al. (2000)
SW403	64 (60-65)	Adbel-Rahman et al. (2000)
SW480	58 (52-59)	Adbel-Rahman et al. (2000)
SW620	48 (45-49)	Adbel-Rahman et al. (2000)
SW837	38 (38-40)	Adbel-Rahman et al. (2000)
LIM1863	80 (66-82)	Adbel-Rahman et al. (2000)
MDA-MB-175	84 (82-89)	ATCC
MDA-MB-468	64 (60-67)	ATCC

A list of cancer cell lines analyzed and estimates of their chromosome number retrieved from the literature.

**Figure 3:**
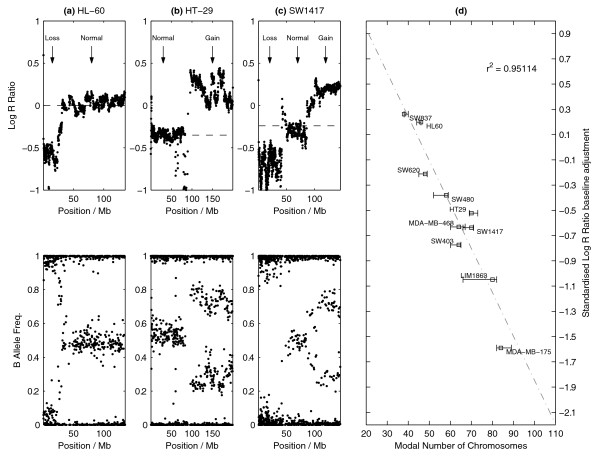
**Estimating baseline Log R Ratio adjustments due to ploidy**. OncoSNP Log R Ratio baseline adjustments (red) for cancer cell lines **(a) **HL60 (Chr10), **(b) **HT29 (Chr3) and **(c) **SW1417 (Chr8). HL60 has a near-diploid karyotype and OncoSNP has correctly identified that no Log R Ratio baseline adjustment is required. HT29 and SW1417 have complex polyploid karyotypes and transformation of the SNP data to a virtual diploid state needs to baseline ambiguity for the Log R Ratio. For example, in (b) and (c), regions of allelic balance with negative Log R Ratios are identified. OncoSNP correctly locates the true baseline level for the Log R Ratio. In **(d) **the estimated Log R Ratio baseline adjustment for the ten cancer cell lines analyzed is found to show a strong linear correlation to the modal chromosome number of each cell line. Baseline adjustments are standardized for comparison against the Log R Ratio level associated with copy number 3 as the SNP data were acquired from different versions of the Illumina SNP array.

Figure 3(a-c) shows examples of the baseline adjustment for three cancer cell lines focusing on selected chromosomes. In each case, OncoSNP adjusts the baseline to center on the regions of allelic balance (BAFs equal to 0.5) corresponding to copy number 2 enabling the correct absolute copy number values to be determined. Note that it is the allele-specific information in the B allele frequencies that inform us of the baseline error, and variation in the intensity-based LRR does not yield this information on its own.

Overall, Figure 3d shows that a strong linear relationship exists with near-diploid cell lines (SW837 and HL60) requiring less baseline adjustment compared to polyploid cell lines. This behavior is encouraging since we might expect the degree of baseline adjustment required to scale linearly with chromosome number. As a result, OncoSNP was able to correctly estimate the chromosome number for each cancer cell line.

### Analysis of normal-cancer cell line mixtures

We applied OncoSNP to three datasets each containing mixtures of normal and cancer cell line DNA. SNP data was also generated in-house for 0:100, 25:75 and 50:50 normal-cancer cell lines mixtures (mixing ratios by mass) for a hypo-diploid (SW837) and triploid (SW403) colon cancer cell line. As paired normal cell lines were not available for these cancer cell lines, we used an non-paired normal DNA sample and filtered out non-compatible SNPs (the filtering method is described in detail in Supplementary methods in Additional file 1) to generate pseudo-paired normal-cancer cell line mixtures. We also analyzed the 0:100, 21:79 and 50:50 mixtures of the HCC1395/HCC1395BL matched normal-cancer cell lines from [24].

**Figure 4:**
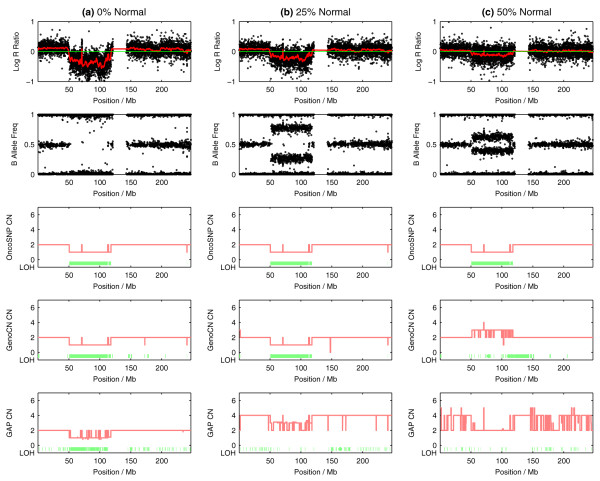
**Example analysis of the normal-cancer cell line (SW837) mixture series**. Copy number and LOH state classifications for chromosome 1 of the colon cancer cell line SW837.

**Figure 5:**
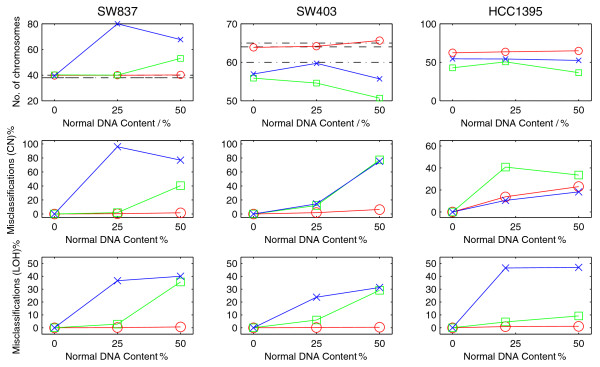
**OncoSNP analysis of three normal-cancer cell line mixture series**. Chromosome number estimates and copy number and LOH state misclassification rates for three normal-cancer cell line mixture series. OncoSNP produces the greatest self-consistency of the three methods tested. Red - OncoSNP, Green - GenoCN, Blue - GAP.

Figure 4 shows results from an analysis of chromosome 1 of the mixture series for SW837. OncoSNP identifies the p-arm deletion successfully in all the samples even as the level of normal contamination increases. GenoCN and Genome Alteration Print (GAP) show less robustness particularly at the higher normal contamination level and, in the case of GAP for the 25:75 mixture, it incorrectly predicts that the sample is tetraploid. Additional plots for all three cell line mixtures are given in Additional file 2. Figure 5 shows that overall, OncoSNP estimates of chromosome number, copy number and LOH from the mixtures remained highly self-consistent even with the addition of the normal DNA and were more robust than the other methods tested. For the colon cancer cell lines, the chromosome numbers predicted by OncoSNP (40 and 64 for SW837 and SW403 respectively) matched known karyotype information (SW837, range 38-40; SW402, range 60 to 65) [36].

Whilst it should be stressed that careful sample preparation should keep normal contamination to a minimum in many real studies of primary tumors, the reliability of OncoSNP, up to 50% tumor purity, is nonetheless reassuring as clinical estimates of tumor purity can be inconsistent with observed genotyping data [25].

### Model comparison

In order to demonstrate the utility of integrating both normal DNA contamination and LRR baseline correction within a single analysis model; we examined SNP data acquired from laboratory generated normal-cancer cell lines mixtures to simulate normal contamination of tumor samples.

The data was analyzed using four variants of our model: a germline model, in which we assume no baseline adjustment is required and no normal DNA contamination exists; a ploidy-only model, in which we perform baseline adjustment only; a normal contamination-only model, where we allow for normal DNA contamination but no baseline adjustment and our full, integrated OncoSNP model. It should be noted that all the model variants we consider are nested within the full model; and are obtained by either fixing parameters or specifying strict prior probability distributions.

**Figure 6:**
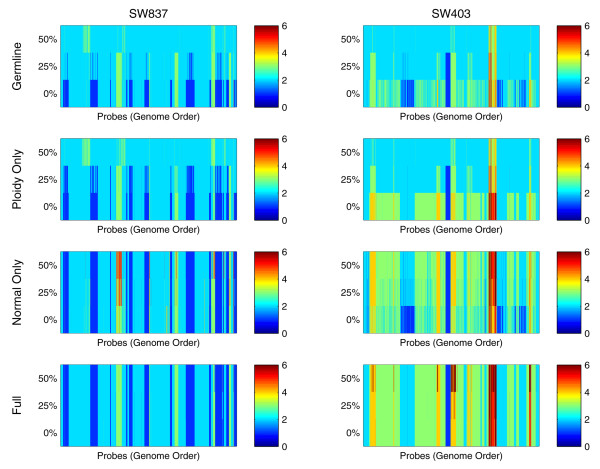
**A comparison of genome-wide copy number estimates using four variants of the OncoSNP model**. Heatmaps are shown for genome-wide copy numbers from four variants of our model: **(i) **Germline model involving no Log R Ratio baseline correction or normal contamination, **(ii) **Ploidy-only model estimation of baseline correction used, **(iii) **Normal-only model estimation of normal DNA contamination used and **(iv) **Full model the complete OncoSNP model incorporating both baseline and normal DNA contamination estimation. The full model is able to accurately reproduce the same copy number profile for both cell lines (SW837/SW403) even in the presence of increasing levels of normal DNA contamination. If normal contamination or baseline correction estimation is not used incorrect copy number profiles maybe given.

Figure 6 shows genome-wide copy number profiles attained from the four variants of our model on the cell line mixtures. The analysis of the hypo-diploid cell line SW837 mixtures showed that the germline- and ploidy-only models, which do not take into account normal DNA contamination, produced substantially different profiles as the level of normal DNA contamination was altered. Only the normal- and full OncoSNP models were capable of reproducing genome-wide copy number profiles consistently with minimal discrepancy.

The analysis of the triploid SW403 cell line mixture series highlights the particular strengths of our model. The correct interpretation of the SNP data requires consideration of the underlying triploid nature of the cancer cell line and the varying levels of normal DNA contamination. As the germline-, normal- and ploidy-only models are only able to compensate for only one of these factors but not both, there are discrepancies in the genome-wide profiles between samples. In contrast, the full OncoSNP model reproduces genome-wide copy number profiles for each mixture sample with relatively greater consistency. These results motivate the utility of inferring both baseline ploidy and normal contamination within an integrated framework since the ploidy status and tumor purity of actual clinical cancer samples are often unknown.

### Microdissected tumor samples

**Figure 7:**
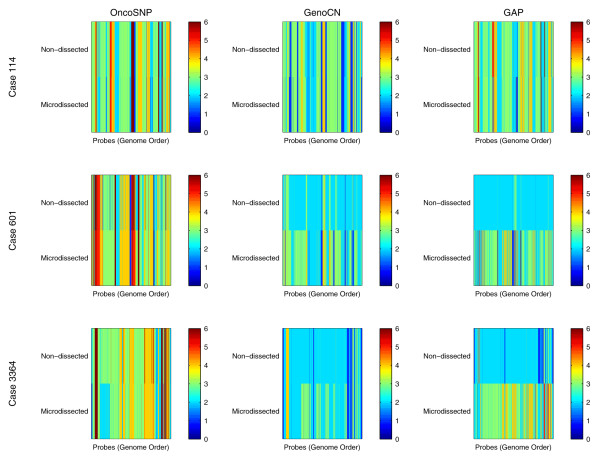
**Genome-wide copy number profiles of primary breast tumors**. Genome-wide copy number profiles for three primary breast tumors (non-dissected and microdissected) using OncoSNP, GenoCN and Genome Alteration Print (GAP).

We validated our approach to determine stromal contamination in an experimental setting by studying SNP data for three primary breast tumors (Cases 114, 601 and 3,364). For each case, we analyzed data acquired from microdissected and non-dissected tumor material such that, in an ideal scenario, predicted copy number and LOH profiles obtained from the two samples should be identical. Visual inspection of the SNP data suggests that all three tumors are triploid and a baseline Log R Ratio adjustment is required. Genome-wide copy number profiles for each material type and case are shown in Figure 7 (more detailed plots are given in Additional file 3). Qualitatively, the genome-wide copy number profiles produced by OncoSNP show the least discrepancy compared to the other methods tested. It should be noted that visual inspection of the SNP data for the non-dissected material for cases 601 and 3,364 suggested that they were highly contaminated by stromal tissue and were reinforced by normal DNA content estimates of 70% and 60% by OncoSNP, compared to 30% and 20% in the microdissected material. The ability of OncoSNP to recover so many gross profile features despite this level of stromal contamination demonstrates its ability to be robust in even the most extreme circumstances. For case 114, the non-dissected and microdissected material were estimated to contain 30% and 10% normal contamination.

Quantitatively, the proportion of SNPs showing copy number classification discrepancies between the microdissected and non-dissected sample analysis were 7.6%, 21.9% and 19.3% for cases 114, 601 and 3,364 respectively. This is compared to 6.4%, 52.1% and 27.0% with GenoCN and 8.5%, 86.2% and 99.0% with GAP. Note that whilst GenoCN showed strong reproducibility for case 114, it misclassified the ploidy in both instances as its operation is limited to diploid tumors.

### Statistical uncertainty

**Figure 8:**
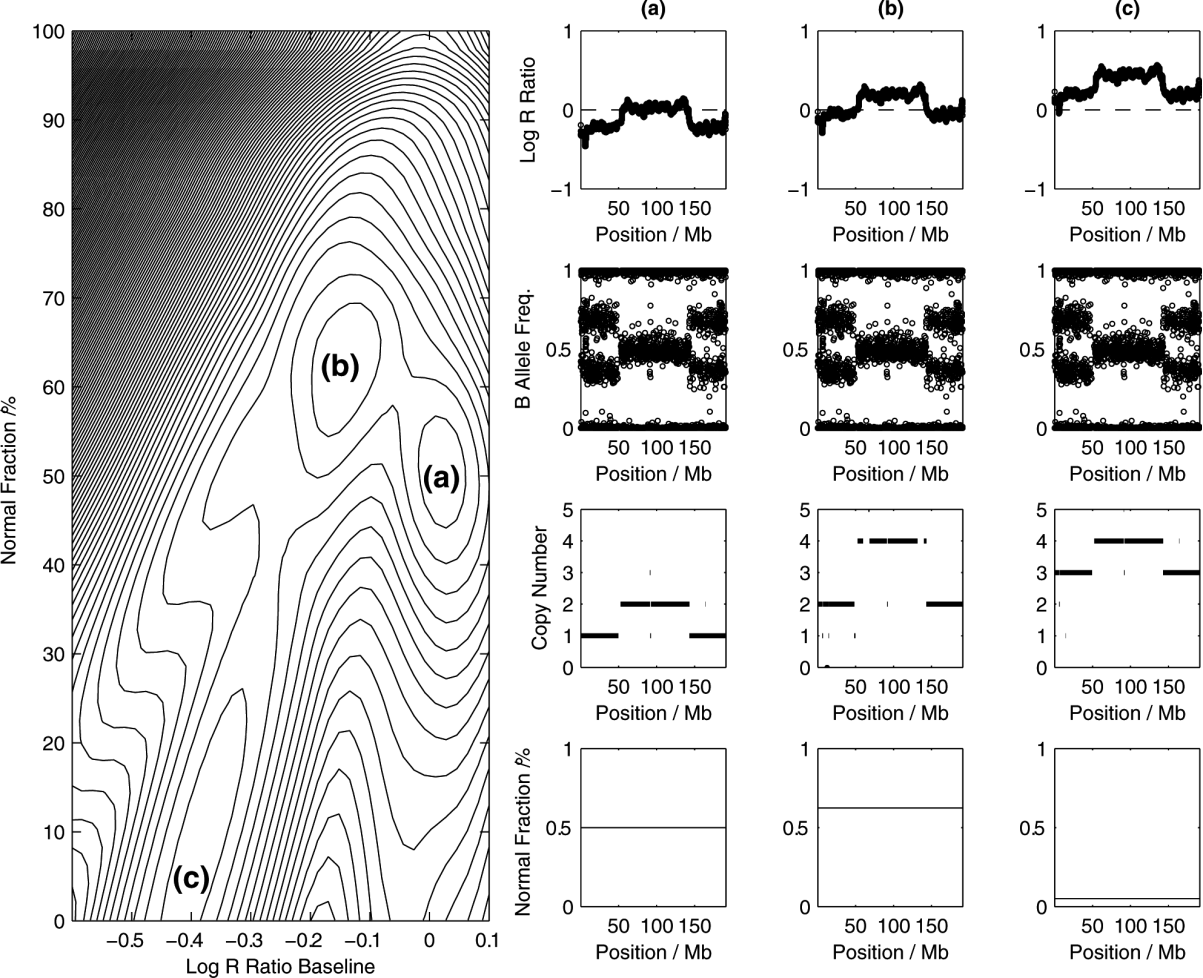
**Analysis of a tumor sample with an unknown ploidy status and normal DNA contamination**. A likelihood contour plot shows that there are three modes each corresponding to an alternative explanation of the SNP data: **(a) **the tumor has near-diploid karyotype and contaminated with 50% normal DNA content, **(b) **the tumor has a tetraploid karyotype with 60% normal DNA content and **(c) **the tumor has a near-triploid karyotype with negligible normal DNA content. The maximum log-likelihood at each mode is very similar.

A feature of our statistical framework is the ability to highlight and explore ambiguity in the interpretation of SNP data from contaminated polyploid tumor samples. Figure 8 shows a likelihood contour plot derived from a cancer sample whose ploidy status and normal DNA content are unknown. The likelihood plot gives the probability of the SNP data associated with different possibilities for the normal DNA content and LRR baseline adjustments. In this example, the likelihood possesses three modes each corresponding to a different, but compatible, biological interpretation of the data. The likelihood associated with each of the three modes is very similar and in the absence of external karyotype information, or prior knowledge of the tumor ploidy or the level of normal DNA contamination, each of these interpretations is entirely plausible. Our statistical model allows us to explore this two-dimensional parameter space enabling each of these data interpretations to be considered in a statistically rigorous manner. In contrast, methods that restrict themselves to consideration of normal DNA contamination or baseline adjustment only will only have access to particular one-dimensional planes which may lead to alternative interpretations of the SNP data being missed. Although we anticipate that many cancers should exhibit a sufficient level of genomic alteration to make the data informative about tumor ploidy and purity, a consideration of alternate ploidy-purity levels maybe an important factor in the characterization of particular cancer sub-types that may not exhibit complex changes.

## Conclusions

The development of our method has been motivated by an on-going genome-wide study of one-thousand paired normal-colorectal cancers. The pro ling of genomic aberrations in these cancers is an important step in identifying genetic abnormalities involved in disease initiation and progression as well as patterns of somatically-acquired alterations associated with particular clinical phenotypes and therapeutic response. The genomic features of colorectal cancer form a particularly useful platform for methods development since colon tumor samples frequently contain normal DNA contamination and there exist at least two well-characterized molecular sub-types: the microsatellite-stable (MSS) and microsatellite-unstable (MSI) groups. MSI colon cancers are associated with a near-diploid karyotype, with comparatively few structural rearrangements; whilst MSS colon cancers are characterized by extensive structural rearrangements and frequently exhibit a triploid or tetraploid karyotype [38]. As our approach considers the combined effects of ploidy changes and tumor heterogeneity jointly within an integrated statistical framework, we have been able to highly automate the process of analyzing SNP data from a large cohort of colon cancers and robustly operate over a range of scenarios posed by each of the molecular sub-types.

Fundamental to the success of our approach is the rigorous exploitation of allele-specific information for estimating normal DNA contamination and tumor ploidy. Historically, one of the key advantages of SNP arrays over aCGH technologies has been the availability of allele-specific information to allow the detection of LOH events. In our method, we have utilized this second axis of information to determine absolute copy number and predict tumor purity that would be challenging to implement with the one-dimensional datasets produced by aCGH alone.

Recently, next generation sequencing (NGS) technologies have proven to be a powerful new force in the toolkit of cancer geneticists allowing cancer genomes to be probe at greater resolutions and more levels of detail than ever before [39-42]. Nonetheless, SNP arrays are likely to remain a useful analysis tool in cancer studies for the foreseeable future as SNP arrays remain more cost- and resource-effective as a means of sampling large numbers of tumors. In addition, as short-read sequencing technologies are not immune to many of the issues that we have discussed. For instance, [42] used pathology review to estimate tumour cellularity in their primary tumour and the brain metastasis and xenograft samples and adjusted sequence read counts accordingly. The integration and reconciliation of SNP data with libraries of short-read sequence data would allow more accurate determination of normal DNA contamination and allow the use of SNP data as a sca old upon which to reconstruct the more detailed and low-level cancer sequence data. It may also be possible to adapt the methods presented here for use for short read sequencing platforms. One possible approach is to model the allele-specific read counts at known SNP locations directly and modify the emission distribution in the Hidden Markov model from a continuous to a discrete distribution (for example Poisson or Negative-Binomial). Alternatively, the existing data model can be maintained and the read counts transformed into near-continuous measures with the Log R Ratio represented as the log ratio of the total read depth and a (local) normalizing constant derived from, say, a matched germline sample and the B Allele Frequency calculated from the ratio of the number of reads containing the B allele to total read depth. However, we would advise that any attempts to implement these techniques for application to sequencing technologies should be supported by extensive control and calibration experiments of the type described in this paper and by previous works.

In conclusion, we have described a novel computational tool (OncoSNP) for genomic copy number and LOH pro ling of heterogeneous tumors using SNP arrays. Using formal statistical modeling we are able to jointly consider a number of complex factors arising in SNP array-based tumor analysis. In a number of experiments, we demonstrated the ability of our method to give consistent results in the presence of both tumor heterogeneity and unknown baseline ploidy using both cancer cell lines and clinical samples. We believe that our method could substantially improve the analysis of tumor SNP data particularly in large studies of clinical samples where there may be exist considerable variation in the underlying genetics as well as factors such as tumor purity and sample quality.

## Materials and methods

### Materials

#### Dilution series

Illumina HumanCNV370-Duo BeadChip Infinium SNP data for dilution series of 12 mixtures of cancer cell line (HCC1395) mixed with its paired normal cell line (HCC1395BL) were downloaded from the NCBI Gene Expression Omnibus accession [GEO:GSE11976]. We excluded chromosome 6 and 16 from analysis due to copy genomic aberrations present in the normal cell line HCC1395BL.

#### Cancer cell lines

Illumina HumanHap300 data for the promyelocytic leukemia cancer cell HL-60 and colon cancer cell line HT-29 were obtained from Illumina, and Human-610 Quad SNP genotyping data for the colon cancer cell lines SW403, SW480, SW620, SW837, SW1417 and LIM1863 were generated at the Ludwig Institute of Cancer Research using standard processing protocols. The genotyping data for breast cancer cell lines MDA-175 and MDA-468 were downloaded from the NCBI Gene Expression Omnibus accession [GEO:GSE18799] [23].

#### Primary breast tumors

Three breast tumors (cases 114, 601 and 3,364) that had not received non-neoadjuvant therapy were analyzed in detail using material derived from microdissection. For each case, material containing pure tumor and pure stroma cells respectively was microdissected and compared to data obtained from surgically obtained material from the same tumors. Case 114 was of Luminal B type (23 mm tumor, moderately differentiated infiltrating ductal carcinoma with an extensive in-situ component. Node +ve, ER +ve (6.8 fm/mg protein), EGFR -ve (7.8 fm/mg protein)). Case 601 (20 mm 30 mm tumor, grade 3 with intraductal in-situ ca. and in filtrating ductal carcinoma, node +ve, ER -ve (1.5 fm/mg protein), Her2 +ve (histoscore of 3), EGFR +ve (histoscore of 208)) was classified as ERBB2 positive based on expression microarray data with a fractional rank of 0.982, Case 3,364 was 25 mm grade 3 infiltrating ductal carcinoma, ER positive (8 fm/mg protein), PR positive (histoscore 8/8), Her2 positive (histoscore 3+, one of ten axillary nodes +ve). For each case, DNA was extracted from microdissected stroma and tumor, as well as the original non-dissected sample and analyzed using Illumina Human-610 Quad SNP arrays applying standard protocols.

#### Data processing

Genome Alteration Print was downloaded [43] and used to analyze all datasets using default settings and the highest ranked copy number and LOH predictions used for comparisons. However, for the cancer cell line dilution series, we re-used the results that had previously been generated by [23] and made available on the aforementioned website.

GenoCN v1.06 was downloaded [44] and used with default settings and stromal contamination settings on for all datasets generated using Illumina Infonaut II SNP arrays. Adjusted GenoCN parameters for the Log R Ratio levels were used for Infonaut HD SNP array processing and in these instances we used the same levels that we specified for OncoSNP. The copy number and LOH predictions from the Viterbi sequence were used for comparisons.

OncoSNP was run on all datasets using 15 EM iterations and with both stromal and intra-tumor heterogeneity options. In all cases, the ploidy prediction with the highest maximum likelihood was chosen and the Viterbi sequence of tumor states used for comparisons. We filtered detected aberrations using a Log Bayes Factor of 30.

### Statistical model

A complete description of our statistical model is provided in Supplementary Information in Additional file 1.

Let ***x***_*i *_denote the tumor state at the *i*-th probe location and (*x*_*i, n*_, *x*_*i, t*_) denote the associated normal and tumor copy numbers. Furthermore, let ***z***_*i *_= (*z*_*i, n*_,*z*_*i, t*_) denote the B allele count for the normal and tumor genotype respectively. The combinations (*z*_*i, n*_, (*x*_*i, n*_) and (z_*i, t*_, *x*_*i, t*_) fully define the normal and tumor genotypes respectively. The tumor state at each probe denotes the allowable combinations of normal-tumor genotypes at that location as shown in Table 1.

Let *π*_0 _denote the normal DNA fraction of the tumor sample due to stromal contamination and $\pi ={\left\{\pi_{i}\right\}}_{i=1}^{n}$ denote the proportion of tumor cells having the normal genotype at each probe. The data $y={\left\{y_{i}\right\}}_{i=1}^{n}$ consists of a set of two-dimensional vectors ***y***_*i *_= [*r_i_*, *b_i_*]' whose elements correspond to the Log R Ratio and B allele frequency respectively.

Given (***x***, ***z***, ***π***, *π*_0_) the data is assumed to be distributed according to a (K + 1)-component mixture of Student t-distributions, where *k_i _*indicates the mixture component assignment of the *i*-th data point,

(1) $$y_{i}|x_{i},z_{i},k_{i},m,\delta ,\;\Sigma =\{\begin{array}{ll} St(m(x_{i},z_{i})+\delta_{k_{l}}^{\left(l_{l}\right)},\sum_{k_{i}}^{\left(l_{i}\right)},\nu ), & k\neq 0,\\ U_{r}(r_{\min},r_{\max})\times {\text{U}}_{b}(0,1), & k=0, \end{array}$$

where $St(\delta_{k}^{\left(l\right)},\Sigma_{k}^{\left(l\right)},v)$ is the probability density function of the Student *t*-distribution with mean $\delta_{k}^{\left(l\right)}$ and covariance matrix $\Sigma_{k}^{\left(l\right)}$ associated with the *k*-th mixture component and the *l*-th genotype class and *v *degrees of freedom. The 0-th component is an outlier class which assumes uniformly distributed data over a specified range.

The elements of the mean vectors ***m***(***x***_*i*_, ***z***_*i*_) = [*m_r_*(***x***_*i*_), *m_b_*(***z***_*i*_, ***x***_*i*_)]' are given by the following:

(2) $$m_{r}(x_{i})=(\pi_{i}(1-\pi_{0})+\pi_{0}){\bar{r}}_{x_{i}{,}_{n}}+(1-\pi_{i})(1-\pi_{0}){\bar{r}}_{x_{x_{i,}{}_{t}}}+\beta_{0}+\beta_{1}g_{i},$$

where *g_i _*is the local GC content at the *i*-th probe location and

(3) $$m_{b}(z_{i},x_{i})=\frac{(\pi_{i}(1-\pi_{0})+\pi_{0})z_{i,n}+(1-\pi_{i})(1-\pi_{0})z_{i,t}}{(\pi_{i}(1-\pi_{0})+\pi_{0})x_{i,n}+(1-\pi_{i})(1-\pi_{0})x_{i,t}}.$$

#### Prior distributions

The prior distribution on the mixture weights is given by a Dirichlet distribution:

(4) $$w^{\left(l\right)}|\alpha \sim Dir(\alpha ),$$

where *α *is a concentration parameter which in the numerical results we used *α *= 1 to give a at prior on the mixture weights.

The prior distributions on the mixture centers and covariance matrices are given by standard conjugate Normal-Inverse Wishart distributions:

(5) $$\delta_{k}^{\left(l\right)}|\tau ,\;\Sigma_{k}^{\left(l\right)}\sim N(0,\tau \;\Sigma_{k}^{\left(l\right)}),\;k=1,\ldots ,\;K,\;l\;=1,2,3,$$

(6) $$\Sigma_{k}^{\left(l\right)}|\gamma ,\;S_{k}^{\left(l\right)}\sim IW(\gamma ,S_{k}^{\left(l\right)}),\;k=1,\ldots ,\;K,\;l\;=1,2,3,$$

where *τ* is a hyperparameter that controls the strength of the prior and IW(*γ*, Λ) denotes the Inverse-Wishart distribution with parameter *γ *and scale matrix Λ.

A beta prior is assumed for the outlier rate,

(7) $$\eta |\alpha_{\eta },\;\beta_{\eta }\sim Be(\alpha_{\eta },\beta_{\eta }),$$

where (*α_n_*, *β_n_*) are hyperparameters associated with the Beta prior. For the numerical results we set these as (1,1) to give a uniform distribution. "

A normal prior is assumed for the local GC content regression parameters,

(8) $$\beta |\lambda_{\beta }\sim N(0,\lambda_{\beta }I_{2}),$$

where ***I***_*p *_is a *p *× *p *identity matrix.

A discrete prior is assumed for the stromal contamination content and intra-tumour heterogeneity levels,

(9) $$p(\pi_{0})=\{\begin{array}{c} \alpha_{\pi_{0}},\;\pi_{0}=0,\\ \beta_{\pi_{0}},\;\pi_{0}>0, \end{array}$$

and

(10) $$p(\pi_{i})=\{\begin{array}{c} \alpha_{\pi },\;\pi_{i}=0,\\ \beta_{\pi },\;\pi_{i}>0, \end{array}\;i=1,\ldots ,n,$$

where in the numerical results we have used *α*_*π*0 _= *β*_*π*0 _= 1 and *α*_*π *_= 1, *β*_*π *_= 2.

The tumor states are assumed to form an inhomogeneous Markov Chain with transition matrix,

(11) $$p(x_{i}|x_{i-1})=\{\begin{array}{c} 1-\rho ,\;\;\;\;\;x_{i}=x_{i-1},\\ \rho ,\;\;\;\;\;\;\;\;\;\;\;x_{i}\neq x_{i-1}, \end{array}$$

where *ρ *= (1/2) (1-exp(-(1/2*L*) (*s*_*i*_-*s*_*i*-1_) and *s_i _*is the physical coordinate of the *i*-th probe and *L *is a characteristic length which we set as *L *= 2,000,000 for the numerical results.

#### Posterior inference

We estimated the unknown model parameters using an expectation-maximization algorithm. Multiple restarts were used to explore different baseline of the Log R Ratio and the baseline with the greatest likelihood was chosen for the calculation of summary statistics.

#### Summary statistics

We used the Viterbi algorithm to extract the most likely sequence of tumors states and for each aberrant segment in the Viterbi sequence we calculated an approximate Bayes Factor (score) of that segment belonging to each of the tumor states. In addition we also recorded the maximum a posteriori estimates of the Log R Ratio baseline adjustment *β*_0 _and the stromal contamination *π*_0_.

### Availability

A MATLAB based implementation (for 64 bit Linux systems) of our software is available for academic and non-commercial use from the associated website [45]. In addition, SNP data analyzed in this paper are also available from this website and from the Gene Expression Omnibus Database under Accession No.[GEO:GSE23785].

## Abbreviations

aCGH: Array-based comparative genomic hybridization; BAF: B Allele Frequency; CNV: Copy number variant; LOH: Loss of heterozygosity; LRR: Log R Ratio; SNP: Single nucleotide polymorphisms.

## Authors' contributions

CY, CCH, SC and JR conceived the method and generated initial ideas and discussions. CY wrote and developed the OncoSNP algorithm. DM, RJ and OS provided bioinformatics analysis and performed genotyping experiments on cancer cell lines. GM, GS, AH and JR provided tumor samples and performed genotyping experiments for the breast cancer analysis. CY, JR, OS and CCH wrote the paper.

## Supplementary Material

Additional file 1 **Supplementary methods**. Detailed description of statistical methodology. Click here for file

Additional file 2 **Genome-wide analysis of three normal-cancer cell line mixtures**. Plots showing genome-wide copy number and LOH analysis for three normal-cancer cell line mixture series. Click here for file

Additional file 3 **Genome-wide analysis of three primary breast tumours**. Plots showing genome-wide copy number and LOH analysis of three primary breast tumours. Click here for file
