# Supplementary material for: A statistical approach for detecting genomic aberrations in heterogeneous tumor samples from single nucleotide polymorphism genotyping data
Source: Genome Biol. 2010 Sep 21;11(9):R92. doi: 10.1186/gb-2010-11-9-r92 (PMC2965384; doi:10.1186/gb-2010-11-9-r92)
Supplement: Additional file 1 — Supplementary methods. Detailed description of statistical methodology. [file gb-2010-11-9-r92-S1.pdf]

A statistical method for detecting genomic aberrations in heterogeneous tumour samples from single nucleotide polymorphism genotyping data

Supplementary Methods

Christopher Yau[1], Dmitri Mouradov[2], Robert Jorissen[2], Stefano Colella[3], Ghazala Mirza[3], Graham Steer[5], Adrian Harris[5], Jiannis Ragoussis[3], Oliver Sieber[2], and Christopher C. Holmes[1,2,5]

[1]Department of Statistics, University of Oxford, Oxford, United Kingdom

[2]Ludwig Institute for Cancer Research, Melbourne, Australia

[3]Wellcome Trust Centre for Human Genetics, University of Oxford, Oxford, United Kingdom

[4]Weatherall institute of Molecular Medicine, University of Oxford, Oxford, United Kingdom

[5]MRC Harwell, Harwell, United Kingdom

September 17, 2010

Contents

1 Introduction **3**

2 Statistical model **5**

2.1 Observation model 5

2.2 Prior distributions 8

3 Posterior Inference **9**

3.1 Conditonal distributions 10

3.2 ECM Updates 11

4 Normalisation of paired normal-tumour data **12**

5 Generating pseudo-paired normal-tumour datasets from non-matching normal and tumour samples **13**

1 Introduction

Illumina SNP genotyping microarrays (SNP arrays) consist of a range of olignucleotide probes designed to target and interrogate the allelic content at a large number (100,000-1,000,0000) of SNPs across the genome. SNP genotyping data consists of a pair of probe measurements (X, Y) whose intensity are proportional to the number of A or B alleles at that SNP. At each probe location from which it is necessary to derive the underlying genotype $g = (z, x)$ where $z \in \{0, \ldots, x\}$ is the number of B alleles and x is the total copy number, e.g. $(1, 2)$ is equivalent to an AB genotype. For humans, x is typically assumed to be equal to two as since the human genome is diploid, therefore $g \in \{AA, AB, BB\}$.

The statistical problem is to make a prediction of g given (X, Y) and many such methods have been developed to achieve this based on clustering using mixture models, regression methods and neural networks. Typically, most genotype calling methods have very high accuracy ($> 99\%$) due to the excellent signal-to-noise ratio of the SNP arrays.

In the presence of copy number variation, x can no longer be assumed to be equal to two, consequently the number of possible genotypes increases beyond the three diploid genotypes, e.g. $g = (1, 4) = AAAB$. Furthermore, the high density of SNP arrays means, copy number alterations tend to be span multiple consecutive probes introducing potential correlation between neighbouring probe measurements.

It is often easier when considering copy number variation to transform SNP data into a different representation. These probe measurements called the Log R Ratio and the B Allele Frequency respectively and are defined (approximately) as follows:

$$R = X + Y, \tag{1}$$

$$r = \log(R/R_{ref}), \tag{2}$$

$$b = \frac{Y}{X + Y} + b_{ref}, \tag{3}$$

where r_{ref} and b_{ref} are constants that adjust for probe-specific biases.

The Log R Ratio (r) measurement given by a particular probe is directly related to the number of copies of DNA captured by the probe (whether they harbour the A or B allele). The B allele frequency (b) is the relative ratio of the number of copies of the DNA capture target DNA sequences containing the B allele to the total DNA copy number.

We use a linear two-component mixture model for the observed Log R Ratio r given by the following

mixture:

$$r_i = \pi_0 \overline{r}_{x_{i,n}} + (1 - \pi_0)\overline{r}_{x_{i,t}} \tag{4}$$

where π_0 is the proportion of normal cells in the sample, $(x_{i,n}, x_{i,t})$ denote the copy numbers for the ith probe of the normal and tumour DNA components and r_x corresponds to the expected Log R Ratio for copy number x. Similarly, for the B allele frequency,

$$b_i = \frac{\pi_0 z_{i,n} + (1 - \pi_0) z_{i,t}}{\pi_0 x_{i,n} + (1 - \pi_0) x_{i,t}} \tag{5}$$

where $(z_{i,n}, z_{i,t})$ denote the number of B alleles in the normal and tumour genotypes.

If the tumour contains J tumour cell types, we can model the observed Log R Ratio and the B allele frequency as a weighted mixture of the J cell types,

$$r_i = \sum_{j=1}^{J} w_j \overline{r}_{x_{i,j}}, \tag{6}$$

$$b_i = \frac{\sum_{j=1}^{J} w_j z_{i,j}}{\sum_{j=1}^{J} w_j x_{i,j}}, \tag{7}$$

where w_j is the proportion of the tumour containing the j-th tumour cell type and $(z_{i,j}, x_{i,j})$ denotes the number of B alleles and the copy number at the i-th probe for the j-th cell type.

It is possible to proceed by performing inference to determine the proportion of each tumour cell type in the sample and the genotypes for each cell type. In general the full inference problem is arguably over-ambitious as it is typical to have access to only first observation sequence consisting on N measurements and there are $2JN+J$ unknowns to be inferred where the number of cell types J is itself an unknown. We can tackle a more tractable problem by making a simplifying assumption. At each locus, we will assume that the tumour cells either possess one (unknown) tumour genotype or retain the normal genotype, $z_{i,j} \in \{z_{i,n}, z_{i,t}\}$ and $x_{i,j} \in \{x_{i,n}, x_{i,t}\}$. This leads to a two-component mixture model involving only $4N + N$ unknown parameters,

$$r_i = \pi_i \overline{r}_{x_{i,n}} + (1 - \pi_i)\overline{r}_{x_{i,t}}, \tag{8}$$

$$b_i = \frac{\pi_i z_{i,n} + (1 - \pi_i) z_{i,t}}{\pi_i x_{i,n} + (1 - \pi_i) x_{i,t}}, \tag{9}$$

where $\pi_i = \sum_{j \in \mathcal{N}_i} w_j$ and $\mathcal{N}_i = \{j : z_{i,j} = z_{i,n}, x_{i,j} = x_{i,n}\}$ is the set of tumour cell types with the normal genotype at the i-th probe. This simplification allows us to avoid the difficult problem of inferring the number of tumour subtypes although we are restricted to scenarios where at any SNP there can only

be one dominant tumour genotype.

We can combine the stromal contamination and the simplified intra-tumour heterogeneity models to obtain the following relationship,

$$r_i = [\pi_0 + (1-\pi_0)\pi_i]\overline{r}_{x_{i,n}} + [(1-\pi_0)(1-\pi_i)]\overline{r}_{x_{i,t}}, \tag{10}$$

$$b_i = \frac{[\pi_0 + (1-\pi_0)\pi_i]z_{i,n} + [(1-\pi_0)(1-\pi_i)]z_{i,t}}{[\pi_0 + (1-\pi_0)\pi_i]x_{i,n} + [(1-\pi_0)(1-\pi_i)]x_{i,t}} \tag{11}$$

where π_i is the proportion of tumour cells retaining the normal genotype at the i-th probe location.

2 Statistical model

2.1 Observation model

Let $\boldsymbol{x} = \{x_i\}_{i=1}^n$ where x_i denotes the tumour alteration at the i-th probe location and $(x_{i,n}, x_{i,t})$ corresponds to the normal and tumour copy numbers. Furthermore, let $\boldsymbol{z} = \{z_i\}_{i=1}^n$ where z_i is an indicator for the normal-tumour genotype combination at the i-th probe where $(z_{i,n}, z_{i,t})$ denotes the number of B alleles in the normal and tumour genotype respectively. The combination of $(z_{i,n}, x_{i,n})$ and $(z_{i,t}, x_{i,t})$ fully define the normal and tumour genotypes. The tumour state and the allowable combinations of normal-tumour genotypes for each tumour state are shown in Table 1. Furthermore, each genotype is associated with one of three genotype classes: HomAA, HetAB and HomBB. We shall model the noise associated with these three classes of genotype separately.

Furthermore, let π_0 denote the normal DNA fraction of the tumour sample due to stromal contamination and $\boldsymbol{\pi} = \{\pi_i\}_{i=1}^n$ denote the proportion of tumour cells having the normal genotype at each probe. The data $\boldsymbol{y} = \{\boldsymbol{y}_i\}_{i=1}^n$ consists of a set of two-dimensional vectors $\boldsymbol{y}_i = [r_i, b_i]'$ whose elements correspond to the Log R Ratio and B allele frequency respectively.

Given $(\boldsymbol{x}, \boldsymbol{z}, \boldsymbol{\pi}, \pi_0)$ the data is assumed to be distributed according to a K-component mixture of Student t-distributions,

$$\boldsymbol{y}_i | x_i, z_i, k_i, \boldsymbol{m}, \boldsymbol{\delta}, \boldsymbol{\Sigma} \sim \begin{cases} \mathrm{St}(\boldsymbol{y}_i; \boldsymbol{m}(x_i, z_i, \pi_i) + \boldsymbol{\delta}_{k_i}^{l_i}, \boldsymbol{\Sigma}_{k_i}^{l_i}), & k_i > 0, \\ \mathrm{St}(\boldsymbol{y}_i; \boldsymbol{0}, \boldsymbol{\Sigma}_\eta), & k_i = 0. \end{cases} \tag{12}$$

where k_i indicates the mixture component assignment of the i-th data point, $\mathrm{St}(\boldsymbol{y}; \boldsymbol{\delta}_k^l, \boldsymbol{\Sigma}_k^l, \nu)$ is the

Tumour Alteration $x_i \rightarrow \{x_{i,n}, x_{i,t}\}$	Genotypes $z_i \vert x_i \rightarrow \{z_{i,n}, z_{i,t}\} \vert x_i$	Genotype Class l_i	Description
$1 \rightarrow \{2, 0\}$	$1 \rightarrow \{0, 0\}$	$1 \rightarrow$ HomA	Homozygous deletion
	$2 \rightarrow \{1, 0\}$	$2 \rightarrow$ HetAB	
	$3 \rightarrow \{2, 0\}$	$3 \rightarrow$ HomB	
$2 \rightarrow \{2, 1\}$	$1 \rightarrow \{0, 0\}$	HomA	Hemizygous deletion
	$2 \rightarrow \{1, 0\}$	HetAB	
	$3 \rightarrow \{1, 1\}$	HetAB	
	$4 \rightarrow \{2, 1\}$	HomB	
$3 \rightarrow \{2, 2\}$	$1 \rightarrow \{0, 0\}$	HomA	Normal copy number
	$2 \rightarrow \{1, 1\}$	HetAB	
	$3 \rightarrow \{2, 2\}$	HomB	
$4 \rightarrow \{2, 3\}$	$1 \rightarrow \{0, 0\}$	HomA	Duplication
	$2 \rightarrow \{1, 1\}$	HetAB	
	$3 \rightarrow \{1, 2\}$	HetAB	
	$4 \rightarrow \{2, 3\}$	HomB	
$5 \rightarrow \{2, 2\}$	$1 \rightarrow \{0, 0\}$	HomA	Copy-neutral LOH
	$2 \rightarrow \{1, 0\}$	HetAB	
	$3 \rightarrow \{1, 2\}$	HetAB	
	$4 \rightarrow \{2, 2\}$	HomB	

Table 1: Tumour States. An example set of allowable normal-germline genotypes for a small selection of tumour states. In actual analysis a larger number of tumour states is required to cover the variety of possible chromosomal alterations that can be observed in tumours.

probability density function of the Student t-distribution with mean $\boldsymbol{\delta}_k^l$, covariance matrix $\boldsymbol{\Sigma}_k^l$ and ν degrees of freedom associated with the k-th mixture component and the l-th genotype class. Note that the genotype class is uniquely defined given (z_i, x_i) and we introduce the label l_i for notational convenience. An outlier class is used $(k_i = 0)$ which assumes the data $\boldsymbol{y}_i$ is distributed according to a Student t-distribution with a large variance Σ_η.

Given the tumour alteration state x_i, the conditional distribution $z_i|x_i$ is given by a Multinomial distribution,

$$z_i|x_i = j, \boldsymbol{q} \sim \text{Mn}(\boldsymbol{q}^j), \tag{13}$$

where $\boldsymbol{q}^j$ are a vector of genotype probabilities associated with the j-th tumour state. We set default values for the elements of $\boldsymbol{q}^j$ as 1/3 for normal-tumour genotypes involving only homozygotes (HomA and HomB) and 1/6 for normal-tumour genotypes involving heterozygotes (HetAB). When performing paired normal-tumour analysis, we use the normal genotype probabilities instead, splitting the probability of heterozygote normal genotypes between tumour genotypes having a normal AB genotype.

Similarly, given the mixture weights $\boldsymbol{w}^l$ of the l-th genotype class, the mixture component assignments are considered to be drawn from a Multinomial distribution,

$$k_i|\boldsymbol{w}^l \sim \text{Mn}(\boldsymbol{w}^l). \tag{14}$$

The elements of the mean vectors $\boldsymbol{m}(x_i, z_i, u_i) = [m_r(x_i, \pi_i), m_b(z_i, x_i, \pi_i)]'$ are given, based on Equation 8 and 9, by the following:

$$m_r(x_i, \pi_i) = [\pi_i(1-\pi_0) + \pi_0]\overline{r}_{x_{i,n}} + (1-\pi_i)(1-\pi_0)\overline{r}_{x_{i,t}} + \beta_0 + \beta_1 g_i, \tag{15}$$

where g_i is the local GC content at the ith probe location. Similarly,

$$m_b(z_i, x_i, \pi_i) = \frac{[\pi_i(1-\pi_0) + \pi_0]z_{i,n} + (1-\pi_i)(1-\pi_0)z_{i,t}}{[\pi_i(1-\pi_0) + \pi_0]x_{i,n} + (1-\pi_i)(1-\pi_0)x_{i,t}}. \tag{16}$$

For copy number (CN) probes targeting monomorphic loci, we use a alternative likelihood based on the

Log R Ratio only:

$$\boldsymbol{y}_i | x_i, z_i, k_i, \boldsymbol{m}, \boldsymbol{\delta}, \boldsymbol{\Sigma} \sim \begin{cases} \mathrm{St}(r_i; m_r(x_i, \pi_i), \sigma^2_{cnv}), & k_i > 0, \\ \mathrm{St}(r_i; \mathbf{0}, \boldsymbol{\Sigma}_\eta), & k_i = 0. \end{cases} \tag{17}$$

where σ^2_{cnv} is the variance associated with CN probes.

2.2 Prior distributions

We use standard Dirichlet priors on the mixture weights of the form:

$$\boldsymbol{w}^l \sim \mathrm{Dir}(\boldsymbol{\alpha}_w), \tag{18}$$

where we typically use set all the elements of the hyperparameter vector $\boldsymbol{\alpha}_w$ to 1 correspondingly to a uniform prior.

The prior distributions on the mixture centres and covariance matrices are given by standard conjugate Normal-Inverse Wishart distributions,

$$\boldsymbol{\delta}^l_k | \tau, \boldsymbol{\Sigma}^l_k \sim \mathrm{N}(\boldsymbol{\delta}^l_k | \mathbf{0}, \tau \boldsymbol{\Sigma}^l_k), \tag{19}$$

$$\boldsymbol{\Sigma}^l_k | \gamma, \boldsymbol{\Sigma}_0 \sim \mathrm{IW}(\boldsymbol{\Sigma}^l_k | \gamma, \boldsymbol{\Sigma}_0), \tag{20}$$

where τ is a concentration parameter and $\mathrm{IW}(\cdot|\gamma, \boldsymbol{\Lambda})$ denotes the Inverse-Wishart distribution with parameter γ and covariance matrix $\boldsymbol{\Delta}$.

A normal prior is assumed for the local GC content regression parameters:

$$\boldsymbol{\beta} | \lambda_\beta \sim \mathrm{N}(\boldsymbol{\beta} | \mathbf{0}, \lambda_\beta \boldsymbol{I}). \tag{21}$$

A beta prior is assumed for the stromal contamination content,

$$\pi_0 | \alpha_{\pi_0}, \beta_{\pi_0} \sim \mathrm{Be}(\pi_0 | \alpha_{\pi_0}, \beta_{\pi_0}), \tag{22}$$

whilst the intra-tumour heterogeneity levels are assumed to be independent at each probe location,

$$\pi_i | \alpha_\pi, \beta_\pi \sim \mathrm{Be}(\pi_i | \alpha_\pi, \beta_\pi), i = 1, \ldots, n. \tag{23}$$

Although it is a trivial extension to include inference on this parameter For the purposes of this paper we shall assume that $\bar{r}$ to be known. This often true in practice since it is possible to perform control experiments involving certain DNA cell lines that contain variable, but known numbers of X chromosomes. Data obtained from these samples can be used to estimate the parameters accurately and reduce the degrees of freedom required for the tumour analysis model.

The tumour states are assumed to form an inhomogeneous Markov chain with transition matrix,

$$p(x_i = j | x_{i-1}) = \begin{cases} 1-\rho, & j = k, \\ \rho, & j \neq k, \end{cases} \tag{24}$$

where

$$\rho = \frac{1}{2}\left[1 - \exp\left\{\frac{1}{2L}(s_i - s_{i-1})\right\}\right],$$

and s_i represents the physical position of the i-th probe and L is a characteristic length parameter.

3 Posterior Inference

Conditional on a value for the stromal contamination π_0, we compute maximum *a posteriori* (MAP) estimates for the model parameters $\theta = \{\eta, \boldsymbol{w}, \boldsymbol{\delta}, \boldsymbol{\Sigma}, \boldsymbol{\beta}\}$ using a expectation-conditional maximisation (ECM) algorithm. We apply the ECM algorithm for a discrete set of values of π_0 between 0 and 1 and find the combination (π_0, θ) that has maximum likelihood.

As the mixture model involves Student t-distributions, we utilise the representation of the Student t-distribution as a scale mixtures of Normal distributions, treating the scaling variables as latent variables in the ECM algorithm,

$$\mathrm{St}(\boldsymbol{y}; \boldsymbol{m}, \boldsymbol{\Sigma}, \nu) = \int_0^\infty \mathrm{N}(\boldsymbol{y}; \boldsymbol{m}, u\boldsymbol{\Sigma}) \mathrm{IG}(u; \nu/2, \nu/2) du \tag{25}$$

where $\mathrm{St}(\boldsymbol{y}; \boldsymbol{m}, \boldsymbol{\Sigma}, \nu)$ is the probability density function of the Student t-distribution with mean $\boldsymbol{m}$, covariance $\boldsymbol{\Sigma}$ and ν degrees of freedom, $\mathrm{N}(\boldsymbol{y}; \boldsymbol{m}, \boldsymbol{\Sigma})$ is the probability density function of the Normal distribution with mean $\boldsymbol{m}$ and covariance $\boldsymbol{\Sigma}$ and $\mathrm{IG}(\cdot)$ is the probability density function of the inverse-Gamma distribution with parameters $(\nu/2, \nu/2)$.

The ECM algorithm obtains updated parameter estimates θ' by maximising the expected complete data

log-likelihood conditonal on the current estimate $\hat{\theta}$:

$$\theta' = \arg\max_{\theta} \mathbb{E}_{\boldsymbol{x},\boldsymbol{z},\boldsymbol{k},\boldsymbol{u},\boldsymbol{\pi}}[\log p(\boldsymbol{y},\boldsymbol{x},\boldsymbol{z},\boldsymbol{k},\boldsymbol{u},\boldsymbol{\pi},\theta)|p(\boldsymbol{x},\boldsymbol{z},\boldsymbol{k},\boldsymbol{u},\boldsymbol{\pi}|\boldsymbol{y},\hat{\theta})], \tag{26}$$

which, under certain regularity conditions, each iteration is guaranteed to increase the likelihood (or posterior probability in this instance).

3.1 Conditonal distributions

In our model, the complete data-likelihood can be decomposed into the following form:

$$\begin{aligned} p(\boldsymbol{y},\boldsymbol{x},\boldsymbol{z},\boldsymbol{k},\boldsymbol{u},\boldsymbol{\pi},\theta) &\propto p(\boldsymbol{y}|\boldsymbol{x},\boldsymbol{z},\boldsymbol{k},\boldsymbol{u},\boldsymbol{\pi},\theta)p(\boldsymbol{k}|\boldsymbol{z},\boldsymbol{x})p(\boldsymbol{z}|\boldsymbol{x})p(\boldsymbol{x})p(\boldsymbol{u})p(\theta), &(27)\\ &= p(\theta)p(\boldsymbol{x})\prod_{i=1}^{n} p(\boldsymbol{y}_i|x_i,z_i,k_i,u_i,\pi_i,\theta)p(\pi_i)p(u_i)p(k_i|z_i,x_i)p(z_i|x_i), &(28)\\ &= p(\theta)p(\boldsymbol{x})\prod_{i=1}^{n} q_{z_i}^{x_i} w_{k_i}^{l_i}\mathrm{St}(\boldsymbol{y}_i;\boldsymbol{m}(x_i,z_i,\pi_i)+\boldsymbol{\delta}_{k_i}^{l_i},\boldsymbol{\Sigma}_{k_i}^{l_i},\nu)p(\pi_i). &(29)\end{aligned}$$

The conditional distribution of the latent variables $(\boldsymbol{u},\boldsymbol{\pi},\boldsymbol{k},\boldsymbol{z},\boldsymbol{x})$ on the current parameter estimates $\hat{\theta}$ can be written as:

$$\begin{aligned} p(\pi_i,u_i,k_i,z_i|x_i,\boldsymbol{y},\hat{\theta}) = p(u_i|\pi_i,k_i,z_i,x_i,\boldsymbol{y},\hat{\theta})p(\pi_i|k_i,z_i,x_i,\boldsymbol{y},\hat{\theta})&\\ \times p(k_i|z_i,x_i,\boldsymbol{y},\hat{\theta})p(z_i|x_i,\boldsymbol{y},\hat{\theta})p(x_i|\boldsymbol{y},\hat{\theta}). &\quad (30)\end{aligned}$$

The marginal distribution of the tumour states $p(\boldsymbol{x}|\boldsymbol{y},\hat{\theta})$ is obtained by integrating over the other latent variables:

$$\begin{aligned} p(\boldsymbol{x}|\boldsymbol{y},\hat{\theta}) &= \sum_{\boldsymbol{z},\boldsymbol{k}}\int_{\boldsymbol{u}}\int_{\boldsymbol{\pi}} p(\boldsymbol{x},\boldsymbol{z},\boldsymbol{k},\boldsymbol{u},\boldsymbol{\pi}|\boldsymbol{y},\hat{\theta})d\boldsymbol{\pi}d\boldsymbol{u}, &(31)\\ &\propto p(\boldsymbol{x})\prod_{i=1}^{n} h(\boldsymbol{y}_i;x_i,\hat{\theta}), &(32)\end{aligned}$$

where

$$\begin{aligned} h(\boldsymbol{y}_i;x_i,\theta) &= \sum_{z_i}\sum_{k_i} \hat{q}_{z_i}^{x_i}\hat{w}_{k_i}^{l_i} F(\boldsymbol{y}_i;\boldsymbol{m}(x_i,z_i)+\hat{\boldsymbol{\delta}}_{k_i}^{l_i},\hat{\boldsymbol{\Sigma}}_{k_i}^{l_i},\nu)p(\pi_i), &(33)\\ F(\boldsymbol{y}_i;\boldsymbol{m}(x_i,z_i)+\hat{\boldsymbol{\delta}}_{k_i}^{l_i},\hat{\boldsymbol{\Sigma}}_{k_i}^{l_i},\nu) &= \int_0^1 \mathrm{St}(\boldsymbol{y}_i;\boldsymbol{m}(x_i,z_i,\pi_i)+\hat{\boldsymbol{\delta}}_{k_i}^{l_i},\hat{\boldsymbol{\Sigma}}_{k_i}^{l_i},\nu)du_i &(34)\end{aligned}$$

Since $\boldsymbol{x}$ is a Markov chain, the marginal probabilities $p(x_i|\boldsymbol{y}, \hat{\theta})$ can be obtained via the forward-backward algorithm.

Conditional on $(x_i, \boldsymbol{y}, \hat{\theta})$ the distribution of the remaining latent variables are given by,

$$p(z_i|x_i, \boldsymbol{y}, \hat{\theta}) = \frac{\sum_{k_i} \hat{q}_{z_i}^{x_i} \hat{w}_{k_i}^l F(\boldsymbol{y}_i; \boldsymbol{m}(z_i, x_i) + \hat{\boldsymbol{\delta}}_k^l, \boldsymbol{\Sigma}_k^l, \nu)}{\sum_{k_i} \sum_{z_i} \hat{q}_{z_i}^{x_i} \hat{w}_{k_i}^l F(\boldsymbol{y}_i; \boldsymbol{m}(z_i, x_i) + \hat{\boldsymbol{\delta}}_k^l, \hat{\boldsymbol{\Sigma}}_k^l, \nu)}, \tag{35}$$

$$p(k_i|z_i, x_i, \boldsymbol{y}, \hat{\theta}) = \frac{\hat{w}_k^l F(\boldsymbol{y}_i; \boldsymbol{m}(z_i, x_i) + \hat{\boldsymbol{\delta}}_k^l, \hat{\boldsymbol{\Sigma}}_k^l, \nu)}{\sum_{k_i} \hat{w}_k^l F(\boldsymbol{y}_i; \boldsymbol{m}(z_i, x_i) + \hat{\boldsymbol{\delta}}_k^l, \hat{\boldsymbol{\Sigma}}_k^l, \nu)}, \tag{36}$$

$$p(\pi_i|k_i, z_i, x_i, \boldsymbol{y}, \hat{\theta}) = \frac{\mathrm{St}(\boldsymbol{y}_i; \boldsymbol{m}(z_i, x_i, \pi_i) + \hat{\boldsymbol{\delta}}_k^l, \hat{\boldsymbol{\Sigma}}_k^l, \nu)}{F(\boldsymbol{y}_i; \boldsymbol{m}(z_i, x_i) + \hat{\boldsymbol{\delta}}_k^l, \hat{\boldsymbol{\Sigma}}_k^l, \nu)}, \tag{37}$$

$$p(u_i|\pi_i, k_i, z_i, x_i, \boldsymbol{y}, \hat{\theta}) = \frac{\nu + 2}{\nu + (\boldsymbol{y}_i - \boldsymbol{m}(z_i, x_i, \pi_i) - \hat{\boldsymbol{\delta}}_{k_i}^{l_i})'(\hat{\boldsymbol{\Sigma}}_{k_i}^{l_i})^{-1}(\boldsymbol{y}_i - \boldsymbol{m}(z_i, x_i, \pi_i) - \hat{\boldsymbol{\delta}}_{k_i}^{l_i})}. \tag{38}$$

3.2 ECM Updates

In order to simplify notation, we define $\overline{u}_i = \int_0^\infty u_i p(u_i|\pi_i, k_i, z_i, x_i, \boldsymbol{y}_i, \hat{\theta}) du_i$, this is the expected value of the scaling variable on the covariance matrix. The integral can be solved analytically.

The ECM update for the outlier rate and mixture weights are given by:

$$\nu = \frac{\sum_{l=1}^3 W_0^l + \alpha_\nu - 1}{\sum_{l=1}^3 \sum_{k=0}^K \left[W_k^l + \alpha_\nu + \beta_\nu - 2\right]}, \tag{39}$$

$$w_j^l = \frac{W_j^l + \alpha_w - 1}{\sum_{k=1}^K \left[W_k^l + \alpha_w - 1\right]}, \tag{40}$$

where $\mathcal{S}(l)$ are the set of genotypes in the l-th genotype class and

$$W_j^l = \sum_{i=1}^n \sum_{x_i} \sum_{z_i \in \mathcal{S}(l)} \int_0^1 \overline{u}_i p(\pi_i, k_i = j, z_i, x_i | \boldsymbol{y}, \hat{\theta}) d\pi_i. \tag{41}$$

The ECM update for the mixture centres and covariances are given by:

$$\boldsymbol{\delta}_j^l = \frac{D_j^l}{W_j^l + \tau}, \tag{42}$$

$$\boldsymbol{\Sigma}_j^l = \frac{\Lambda_j^l + \tau[\boldsymbol{\delta}_j^l][\boldsymbol{\delta}_j^l]' + \gamma \boldsymbol{\Sigma}_0}{W_j^l + \gamma - 2}, \tag{43}$$

where

$$D_j^l = \sum_{i=1}^{n} \sum_{x_i} \sum_{z_i \in \mathcal{S}(l)} \int_0^1 \overline{u}_i [\boldsymbol{y}_i - \boldsymbol{m}(z_i, x_i, \pi_i)] p(\pi_i, k_i = j, z_i, x_i | \boldsymbol{y}, \hat{\theta}) d\pi_i, \tag{44}$$

$$\Lambda_j^l = \sum_{i=1}^{n} \sum_{x_i} \sum_{z_i \in \mathcal{S}(l)} \int_0^1 \overline{u}_i [\boldsymbol{y}_i - \boldsymbol{m}(z_i, x_i, \pi_i)][\boldsymbol{y}_i - \boldsymbol{m}(z_i, x_i, \pi_i)]^T p(\pi_i, k_i = j, z_i, x_i | \boldsymbol{y}, \hat{\theta}) d\pi_i, \tag{45}$$

The ECM updates for $\boldsymbol{\beta}$ are obtained by solving the following linear equations:

$$\sum_{i=1}^{n} \sum_{x_i} \sum_{z_i} \sum_{k_i} \int_0^1 \overline{u}_i \psi(\pi_i, k_i, z_i, x_i) p(\pi_i, u_i, k_i, z_i, x_i | \boldsymbol{y}, \hat{\theta}) d\pi_i = \lambda_\beta \beta_0, \tag{46}$$

$$\sum_{i=1}^{n} \sum_{x_i} \sum_{z_i} \sum_{k_i} \int_0^1 g_i \overline{u}_i \psi(\pi_i, k_i, z_i, x_i) p(\pi_i, u_i, k_i, z_i, x_i | \boldsymbol{y}, \hat{\theta}) d\pi_i = \lambda_\beta \beta_1, \tag{47}$$

where

$$\psi(\pi_i, k_i, z_i, x_i) = 2(\Sigma_{11}^{-1})_{k_i}^{l_i} [r_i - \overline{r}(\pi_i, x_i) - \beta_0 - \beta_1 g_i] - [(\Sigma_{12}^{-1})_{k_i}^{l_i} + (\Sigma_{21}^{-1})_{k_i}^{l_i}][b_i - \overline{b}(\pi_i, z_i, x_i)] \tag{48}$$

Note that, in our implementation, the integrals over the intra-tumour heterogeneity coefficients π_i are calculated numerically using quadrature on a grid of 10 points.

4 Normalisation of paired normal-tumour data

One of the major challenges in removing array-specific noise from tumour data is that the noise is confounded by the presence of tumour-specific genomic aberrations. However, when data for paired normal and tumour samples are acquired on the same SNP array (such as the multi-sample format Illumina Duo or Quad arrays), we can treat the normal sample as a "noise" template and use it to remove array-specific noise from the tumour sample.

Let (x_i, y_i) represent the Log R Ratio of the i-th probe for the normal (x) and tumour (y) sample in a window of n probes in the neighbourhood of the j-th probe. We represent the relationship between the Log R Ratios of the normal and tumour samples in the window using the following linear model:

$$y_i = \boldsymbol{X}_i \boldsymbol{\beta}_j + \epsilon_i, \; i = 1, ..., n, \tag{49}$$

where $\boldsymbol{X}_i = [1 \; x_i]$ is the i-th row of the $n \times 2$ design matrix, $\boldsymbol{\beta}_j$ is a 2×1 column vector of probe-

specific regression coefficients and ϵ_i is independent, identically distributed noise which follows a Student t-distribution with ν degrees of freedom and variance σ^2.

We can derive a maximum likelihood estimator for the regression coefficients using an expectation-maximisation algorithm which iterates between the following two operations:

$$\boldsymbol{\beta}_j = (\boldsymbol{X}^T\boldsymbol{W}\boldsymbol{X})^{-1}\boldsymbol{X}^T\boldsymbol{W}\boldsymbol{y}, \tag{50}$$

$$\sigma^2 = \frac{1}{n}(\boldsymbol{y} - \boldsymbol{X}\boldsymbol{\beta}_j)^T\boldsymbol{W}(\boldsymbol{y} - \boldsymbol{X}\boldsymbol{\beta}_j). \tag{51}$$

The diagonal elements of the $n \times n$ weight matrix $\boldsymbol{W}$ are given by,

$$E(1/V_i|y_i, \boldsymbol{\beta}_j, \sigma^2, \nu) = \frac{1}{\nu\sigma^2 + (y_i - \boldsymbol{X}_i\boldsymbol{\beta}_j)^2}, \ i = 1, \dots, n. \tag{52}$$

The corrected Log R Ratio value for the j-th probe of the tumour is given by:

$$\tilde{y}_j = y_j - \beta_{j,1}x_j. \tag{53}$$

It is necessary to replace extreme values of the Log R Ratio x for the normal samples with neighbourhood averages in order to avoid artefacts in the corrected Log R Ratios for the tumours $\tilde{y}$. Furthermore, instead of performing the regression for every probe and using overlapping windows, we can apply the analysis using non-overlapping windows and use the regression coefficients of each window to obtain corrected Log R Ratios for every probe in the window. This can give considerable computational speed-up with little difference in output quality.

5 Generating pseudo-paired normal-tumour datasets from non-matching normal and tumour samples

Matched normal DNA samples for cancer cell lines are often unavailable and limits our ability to generate normal-cancer cell line mixtures in order to test methods, such as OncoSNP. Ideally, we would like to use readily obtainable unmatched normal DNA but the lack of "relatedness" will give rise to many inconsistencies between the genotypes of the normal and cancer cell line which will prove problematic.

In the following we propose a two-stage filtering method to identify and select those SNPs where such

inconsistencies arise so that they maybe remove from the data acquired from mixtures of non-matched normal-cancer cell lines. The result is pseudo-matched mixed normal-cancer cell line datasets that can be used for testing algorithmic performance. We begin by assuming that we have genotypes available for the non-matching normal DNA sample and pure cancer cell lines. For the cancer cell line, an accurate genotype classification is not required, we only need to know whether the SNP is homozygous for allele A or B or heterozygous and contains at least one contribution from each allele.

Filter Stage I: Finding Genotype mismatch. As the normal and cancer cell line are not matched, there will be many thousands of SNPs where the genotypes are incompatible. For example, the genotype in the cancer cell line might be homozygous A but the non-matching normal sample could have a BB genotype. This is not possible without a somatic mutation involving a nucleotide substitution and, whilst such mutations can occur in cancer genomes, the probability that a mutation occurs and is also coincident with a SNP targetted by the SNP array is extremely small. Table 2 shows all the possible matching and non-matching normal-cancer genotype combinations. The first filtering stage identifies all SNPs with incompatible normal-cancer genotypes and discounts those SNP from consideration in the mixed normal-cancer cell line samples.

		Normal		
		AA	AB	BB
Cancer	Hom. A	✓	✓	×
	Het.	×	✓	×
	Hom. B	×	✓	✓

Table 2: Matching (✓) and non-matching (×) normal-cancer genotype combinations.

Filter Stage II: Identifying mismatched autozygosity patterns. Genome-wide differences in the autozygosity patterns between the non-matching normal and cancer cell line can also lead to artefacts in the mixed samples. This is because runs of homozygous genotypes in the cancer cell line which are due to autozygosity are actually loss-of-heterozygosity (LOH) events relative to the non-matching normal sample which may possess heterozygous genotypes in these regions. The differences in autozygosity patterns therefore lead to an inflated number of pseudo-somatic LOH events. In order to reduce the number of these false LOH events, we need to identify autozygosity

We assume that, at each SNP, the (unobserved) state (x_i) of the i-th SNP can be classified as either a normal, autozygosity or LOH region. The states are assumed to show a dependence structure which we model using a three-state Markov model with transition matrix π and state-conditional emission probabilities for the observations are given by ϕ.

We use an expectation-maximisation algorithm to learn the HMM parameters. We initialise the transition

State, x	Description	$\phi(AA\|x)$	$\phi(AB\|x)$	$\phi(BB\|x)$	$\phi(NC\|x)$
1	Normal	$(1-\nu)/3$	$(1-\nu)/3$	$(1-\nu)/3$	ν
2	Autozygosity	$(1-\nu)/2$	0	$(1-\nu)/2$	ν
3	LOH	$(1-\nu)/2$	0	$(1-\nu)/2$	ν

Table 3: Initial values for the state-conditional emission probabilities.

matrix π to the following,

$$\pi = \begin{pmatrix} 1-\rho_0 & \rho_0/2 & \rho_0/2 \\ \rho_a/2 & 1-\rho_a & \rho_a/2 \\ \rho_l/2 & \rho_l/2 & 1-\rho_l \end{pmatrix} \tag{54}$$

where $(\rho_0 = 0.001, \rho_a = 0.01, \rho_l = 0.001)$ are the transition probabilities out of the normal, autozygosity and LOH states respectively. The state-conditional emission probabilities are initialised to the values given in Table 3 with $\nu = 0.01$.

Figure 1 shows the effects of each filtering stage. The use of the genotype mismatch filter removes SNP which exhibit hard genotype inconsistencies between the cancer cell line and the non-matching normal sample. The autozygosity filter then removes the SNPs in autozygosity regions in the cancer cell lines. The result is a normal-cancer cell line mixture series that mimics a real mixture series using matched cell lines.

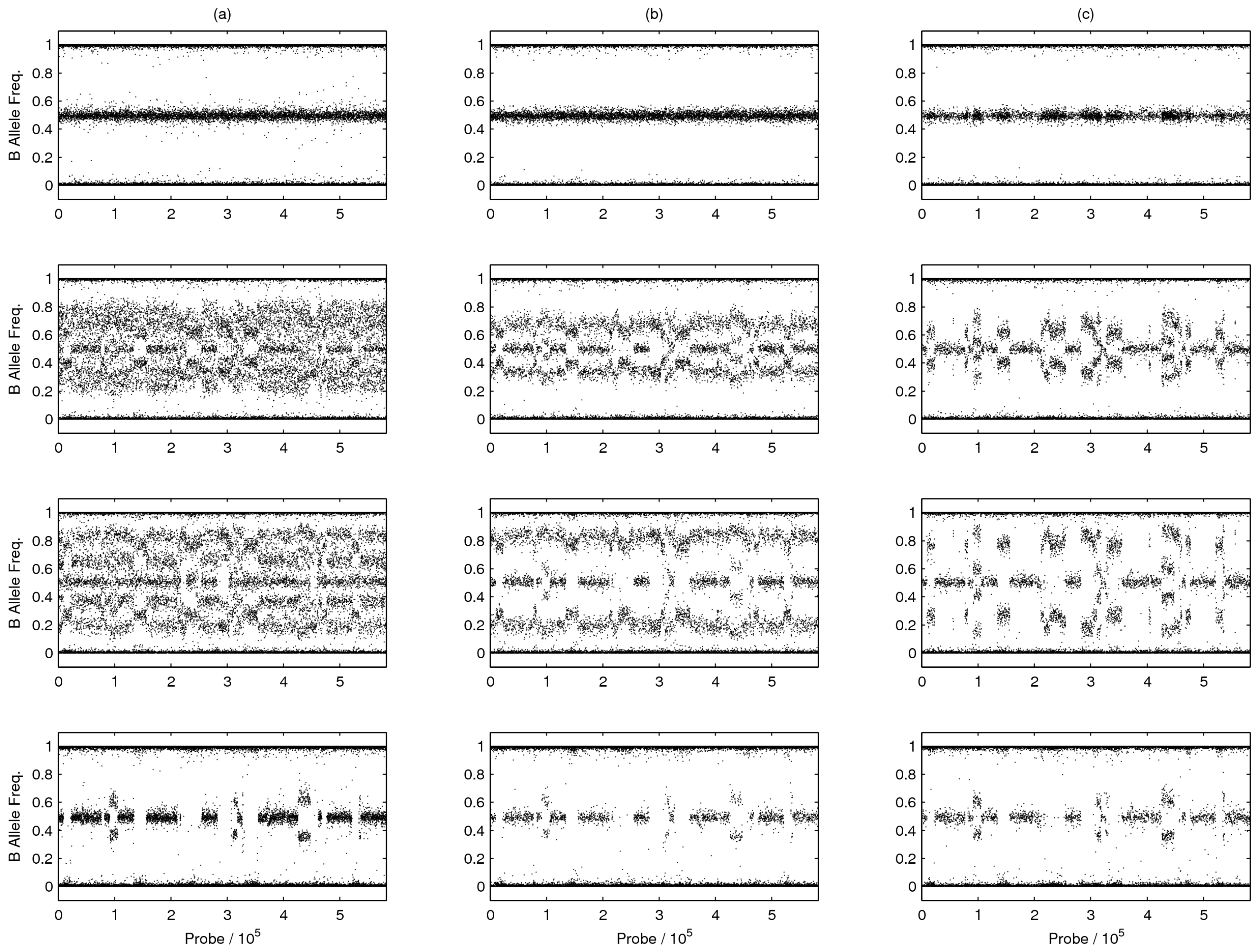

Figure 1: A filtering process for generating pseudo paired normal-tumour data. Normal:Cancer Cell Line (SW837) Mixtures (top to bottom) 100:0, 50:50, 25:75 and 0 :100. (a) Original Data, (b) Genotype Mismatch filter (Stage I) and (c) Autozygosity filtered (Stage I and II).
